# Digital Twins for Hospital and Healthcare Operations: A Systematic Review of Resource Allocation, Infection Control, and Workflow Optimization

**DOI:** 10.3390/bioengineering13091072

**Published:** 2026-09-15

**Authors:** Nesma Abd El-Mawla, Mohamed Shehata, Mostafa A. Elhosseini

**Affiliations:** 1 Department of Computer Science and Engineering, Faculty of Computer Science and Engineering, New Mansoura University, New Mansoura 35712, Egypt; 2 Computer Science Program, James C. Bowling School of Business, Midway University, Midway, KY 40347, USA; 3 College of Computer Science and Engineering, Taibah University, Yanbu 46411, Saudi Arabia; 4 Computers Engineering and Control Systems Department, Faculty of Engineering, Mansoura University, Mansoura 35516, Egypt

**Keywords:** digital twins, healthcare operations, AI, IoT, resource allocation, infection control, workflow optimization

## Abstract

The incorporation of Digital Twins (DT) into the healthcare industry marks a revolution in terms of adopting a more proactive and personalized approach towards patient care. The increasing complexity of technological tools employed within the healthcare environment leads to assessing the potential impacts of these digital models in collaboration with AI and IoT for increased efficiency and improved results. In this context, this study offers a systematic review of existing research regarding DTs in the field of healthcare, with specific consideration of hospital applications. An extensive literature search was performed within the Scopus database for peer-reviewed publications during the period from 2021 to 2026. Following a demanding screening process, 70 relevant articles were found that fulfilled the selection criteria. The review shows an emerging trend towards the application of AI-based Digital Twins in the real-time monitoring, predictive maintenance of medical devices, and planning surgeries. The paper analyses several key characteristics of healthcare DTs, including their design and architecture, and the benefits they generate. It also presents the challenges related to data integration and ethics surrounding virtual health models and recommendations for future research. In conclusion, this review demonstrates the revolutionary role of AI- and IoT-enabled Digital Twins in the transformation of hospitals’ infrastructures. This paper summarizes the latest developments and gaps in this field and offers a starting point for further research in this area.

## 1. Introduction

**Figure 1:**
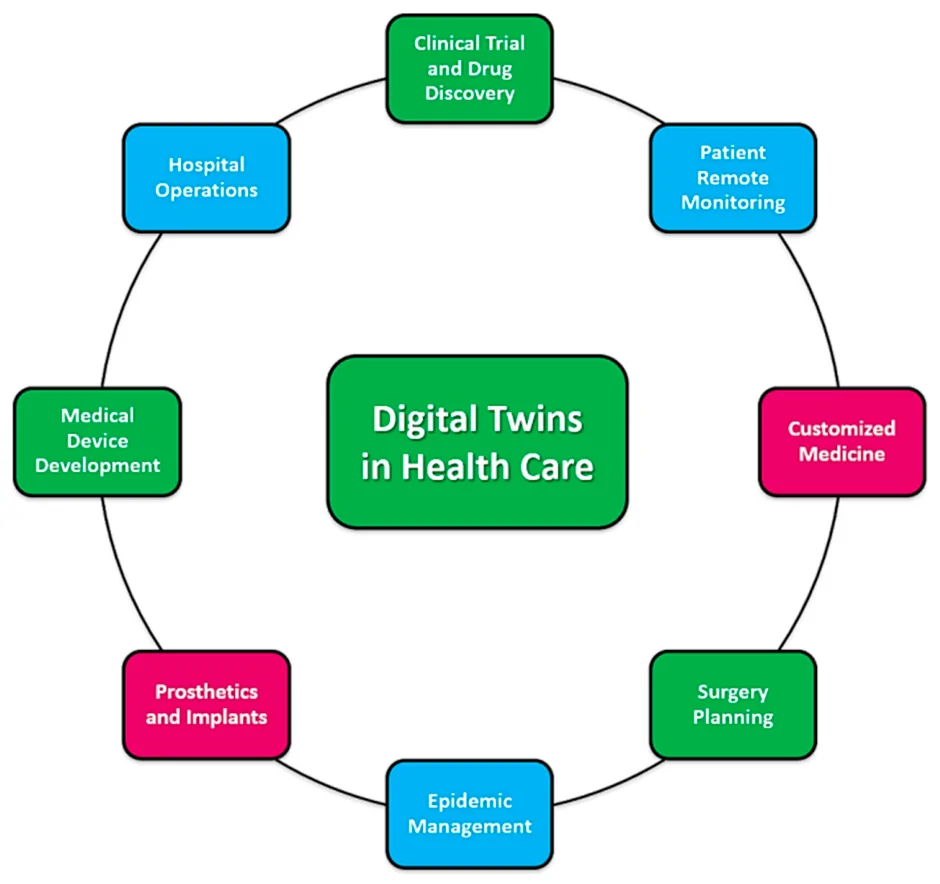
Key application domains of Digital Twin technology in modern healthcare and medical research.

Digital Twin [1] technology can be characterized as the virtual reflection of an actual item or a system that is updated with real-time data while relying on simulation, machine learning, and reasoning to enhance decision-making. In the context of healthcare, it goes beyond modeling of individual patients to encompass operations at the level of entire facilities. Nowadays, it has many different applications [2,3,4,5], as illustrated in Figure 1.

Healthcare Digital Twin is an environment where (i) efficient allocation of beds and staffing takes place, (ii) patient movements are controlled in real-time, (iii) infection threats are predicted and mitigated, and (iv) decisions are made using predictive analysis tools.

In response to the unprecedented situation in the global healthcare system during the pandemic in 2020, there was an increased need for finding new ways to deal with surge capacity and provide protection to personnel. As a result, there appeared a range of non-traditional applications of AI and IoT that would help decrease any bottlenecks. The use of artificial intelligence in a digital twin model comes with several benefits [6,7,8,9,10], namely (i) automation of the scheduling process to facilitate healthcare professionals’ work, (ii) insights into large volumes of data produced by hospitals, and (iii) integration of various medical assets.

The reason behind conducting this study is due to the increasing pressure faced by healthcare management globally because of increased costs, aging populations, and the need for flexibility during public health crises. Conventional management practices typically face challenges with inefficient use of resources and an inability to adjust to changes. The use of Digital Twin (DT) with the help of artificial intelligence and IoT allows for more efficient and flexible operations through the ability to simulate potential outcomes with zero risk and to make decisions based on simulations. One problem with current DT implementations is that they tend to be “fragmented,” meaning that most of the implementations deal with one unit (MRI appointments, patient monitoring, tracking a certain illness, etc.) and not as an integrated DT solution throughout the entire facility. Additionally, a need for prescriptive analytics exists because the current state of healthcare DT implementations stops with descriptive visualizations without implementing self-governance to make better decisions automatically based on complex hospital operations. Lastly, “interoperability barriers” in terms of heterogeneous data integration, including EHRs, patient devices (wearables), building management systems, etc., need to be addressed.

According to the current bibliometric trend, the use of DTs in healthcare is an emerging area of study. As evident from the conceptual framework of contemporary AI, the next step in moving towards smart hospitals is shifting from reactive to predictive models.

### 1.1. Paper Contributions

The distinguishing feature of this systematic review, in comparison with other studies conducted in the field of healthcare DTs, is the shift in the approach from technical analysis towards a sociotechnical one by closing the Gap between Sociology and Technology. Although in conventional reviews the efficiency of the AI algorithm and the technology of DTs are in the spotlight. The objective of the paper is to attract the attention of the world to DTs for hospitals and health care operations and focus on sustainability aspects over AI and IoT. The paper focuses on projects and research work related to DTs and their future research directions. The current paper provides a detailed overview of DTapplications in the healthcare sector. The main contributions of the paper include:

1. A Systematic Review of DTs for hospital and healthcare operations by discussing the topic is presented in the paper, covering its theoretical foundation and applications.
2. Presenting a detailed discussion on metrics, standards, benchmark simulators, and datasets involved in evaluating DTs in hospitals and healthcare technologies is discussed in this paper.
3. A Detailed Discussion of DTs for Hospital and Healthcare Operations Using AI and IoT Systems through an extensive discussion on DTs through AI and IoT technologies is presented in the paper.
4. Closing the Gap between Sociology and Technology combines technical efficiency with such human factors as patients’ rights, sociological issues, and self-regulation of the system. This study offers a prospective framework, which enables stakeholders to assess the sustainability of DTs by balancing the benefits of service provision (for example, cost reduction, high-quality treatment) and human concerns that, according to the authors, are overlooked in modern literature.
5. A forward-looking view on challenges and future research directions by describing upcoming research trends and directions to outline the necessary steps for further advancements in DT applications in hospital and healthcare operations.
6. Attention to Ethical Aspects of Sustainability, as there is identification and analysis of the ethical aspects of AI-based healthcare systems to ensure that the analysis considers aspects not only of the technical efficiency of the DT but also of its responsibility and sustainability.

**Figure 2:**
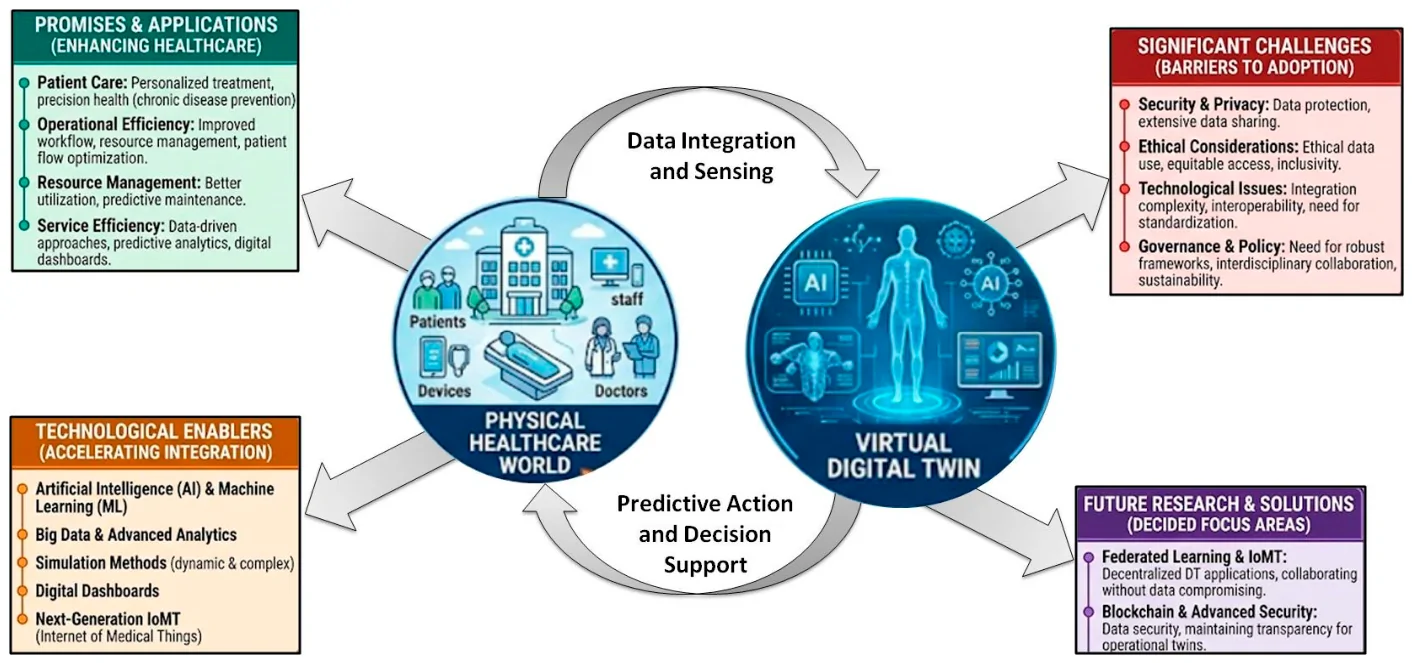
Comprehensive overview of Digital Twins’ ecosystem in healthcare.

By providing a detailed and insightful analysis of these key areas, this paper aims to stimulate further research and innovation in the field of DTs for hospital and healthcare operations, ultimately contributing to a more sustainable future as shown in Figure 2.

### 1.2. Paper Organization

The rest of this review paper is structured as follows: Section 2 introduces the theoretical basis of DTs in healthcare by discussing their definitions and the architecture of AI, IoT, and medical data integration. Section 3 describes the research methodology and explains the bibliometric analysis carried out. Section 4 compares the current study with previous review papers to justify the uniqueness of this study and research gaps. Section 5 investigates the major applications of digital twins in hospitals and healthcare services in terms of resource allocation, infection control, workflow optimization, and smart infrastructure management. Section 6 discusses the performance evaluation of these systems and benchmarking frameworks. Lastly, Section 7 analyses the critical challenges, open questions, and future research directions related to security, interoperability, and Federated Learning, among other issues. Finally, the paper is concluded.

## 2. Definition and Concepts

DT in healthcare [11,12] is defined as a dynamic, virtual representation of a physical entity such as a patient, a medical device, or an entire hospital facility maintained through a continuous, bidirectional data exchange. Unlike traditional static models, a healthcare DT functions as a living replica that synchronizes real-time data from its physical counterpart to enable monitoring, simulation, and optimization. This concept has evolved from industrial “Industry 4.0” applications to become a cornerstone of Precision Medicine and smart hospital management, where it supports predictive interventions and individualized care strategies [13,14,15].

### 2.1. Architecture and Components

**Figure 3:**
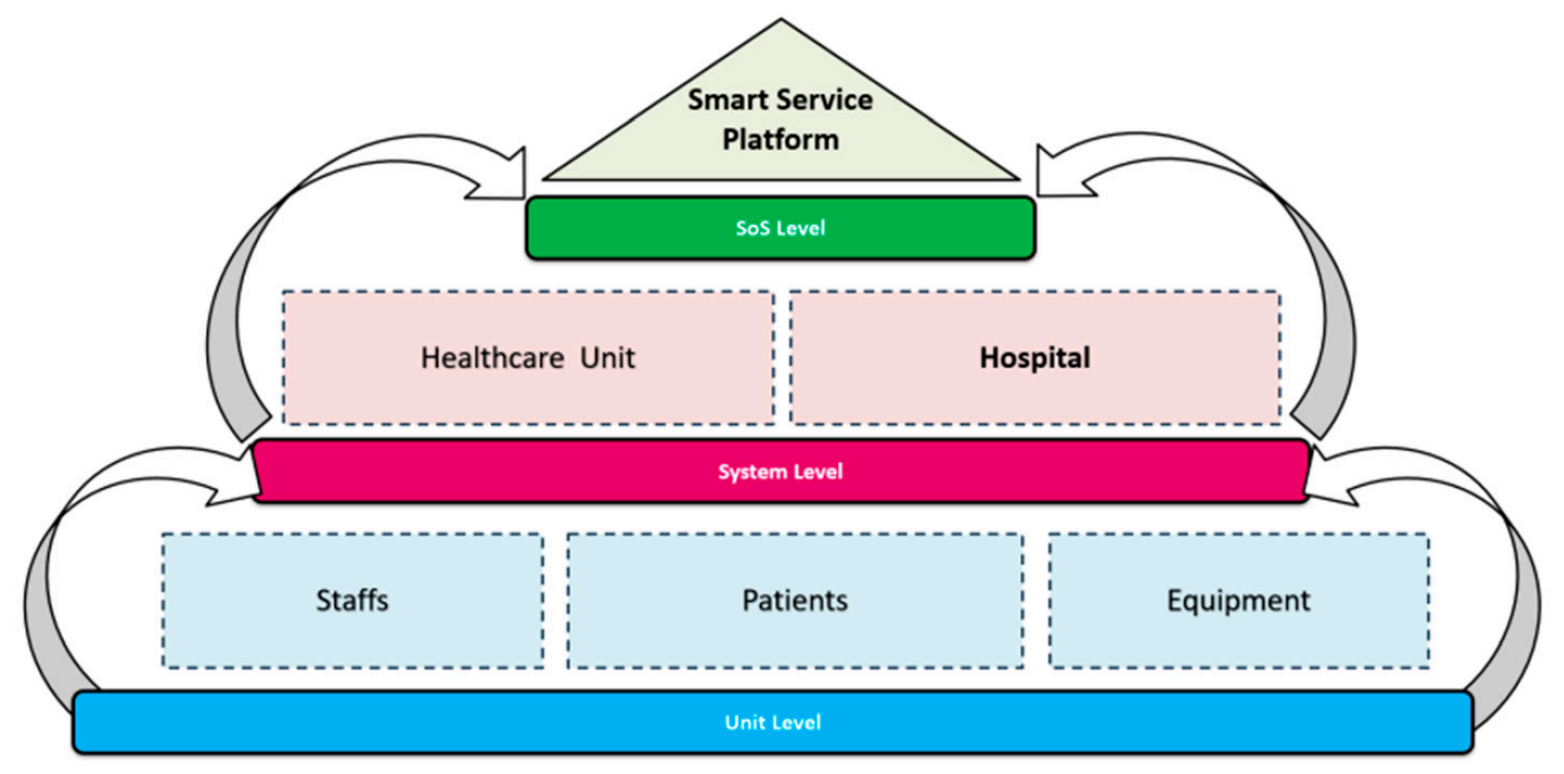
Multi-level operational hierarchy of a smart healthcare service platform, detailing the relationship between the Unit, System, and SoS levels.

The architecture of a healthcare Digital Twin generally consists of three interconnected layers as seen in Figure 3. The system architecture consists of three operational layers, beginning with the Unit Layer, which gathers real-time data and events from key participants and resources, such as doctors, patients, and advanced equipment. These data accumulate into the System Layer, where each Healthcare Unit and Hospital operates as digital representations of themselves to streamline their inner workings. The topmost layer is known as the System of Systems (SoS) Layer, which coordinates between different healthcare systems using a smart services platform. This allows for a smooth transfer of information in both directions, as illustrated by the arrows.

### 2.2. Types of Healthcare DTs

Digital Twins are divided into different types according to their applications: (i) Patient Digital Twin (PDT): A digital twin that contains virtual copies of physiological and biological information of a person, useful in providing personalized care, managing chronic diseases, and conducting simulations of clinical trials. (ii) Operational/Hospital Digital Twins: Digital twins aimed at optimizing operational efficiency in hospitals by improving the allocation of hospital resources and ensuring that infection control guidelines are met. (iii) Cognitive Digital Twins: An advanced form of digital twins that uses machine learning algorithms to autonomously make decisions.

**Figure 4:**
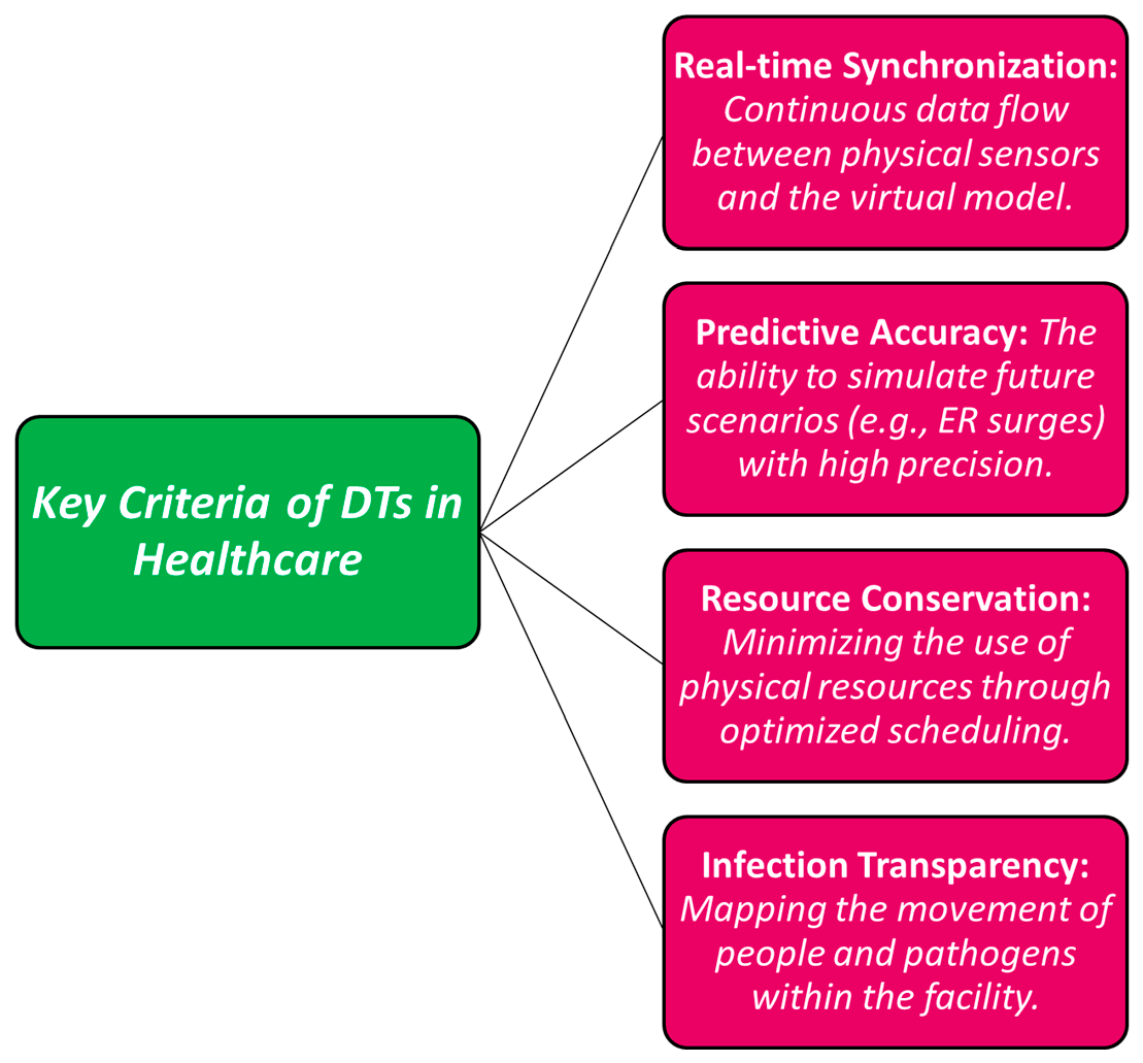
Key criteria for DTS of smart healthcare.

Further, for a hospital Digital Twin to be effective, it must meet the following criteria as shown in Figure 4: (i) Real-time Synchronization. (ii) Predictive Accuracy. (iii) Resource Conservation. (iv) Infection Transparency.

### 2.3. AI, IoT, and Data Integration

**Figure 5:**
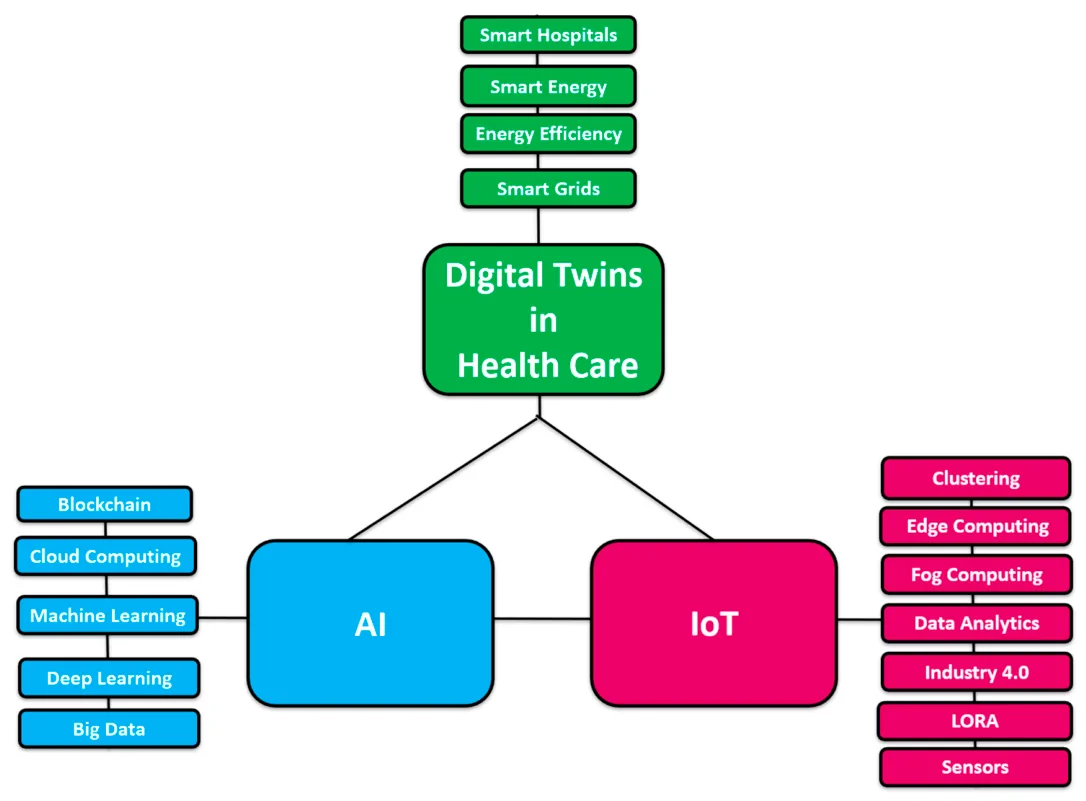
Key Terms of AI and IoT in DTs and Healthcare.

In terms of the role of AI in this technology ecosystem, several technologies can be mentioned, including Machine Learning, Deep Learning, Big Data Analytics, as well as Blockchain and Cloud Computing. At the same time, IoT technology plays an equally important role in this ecosystem and is related to the connection of clinical solutions with the overall concept of Industry 4.0 through Sensors, LORA, and Edge/Fog Computing technologies. Thus, Digital Twins help to establish Smart Hospitals, providing efficient Smart Energy management and Energy Efficiency, which includes implementing Smart Grids in the modern healthcare setting. Figure 5 presents the technology ecosystem behind the development of Digital Twins in Healthcare, where AI and IoT play important roles. The effectiveness of a healthcare DT relies on the deep integration of several core technologies:

- Internet of Things (IoT): Provides the vast array of networked sensors and wearable devices required for continuous data harvesting from patients and environments.
- Artificial Intelligence (AI): Serves as an analytical engine, employing deep learning algorithms (such as CNNs and LSTMs) to process complex clinical and operational data for predictive insights.
- Data Integration Challenges: A critical hurdle remains the fusion of heterogeneous data sources, including Electronic Health Records (EHRs), real-time sensor streams, and building management systems, which must be standardized to ensure model accuracy and scalability.

## 3. Research Methodology and Bibliometric Analysis

**Figure 6:**
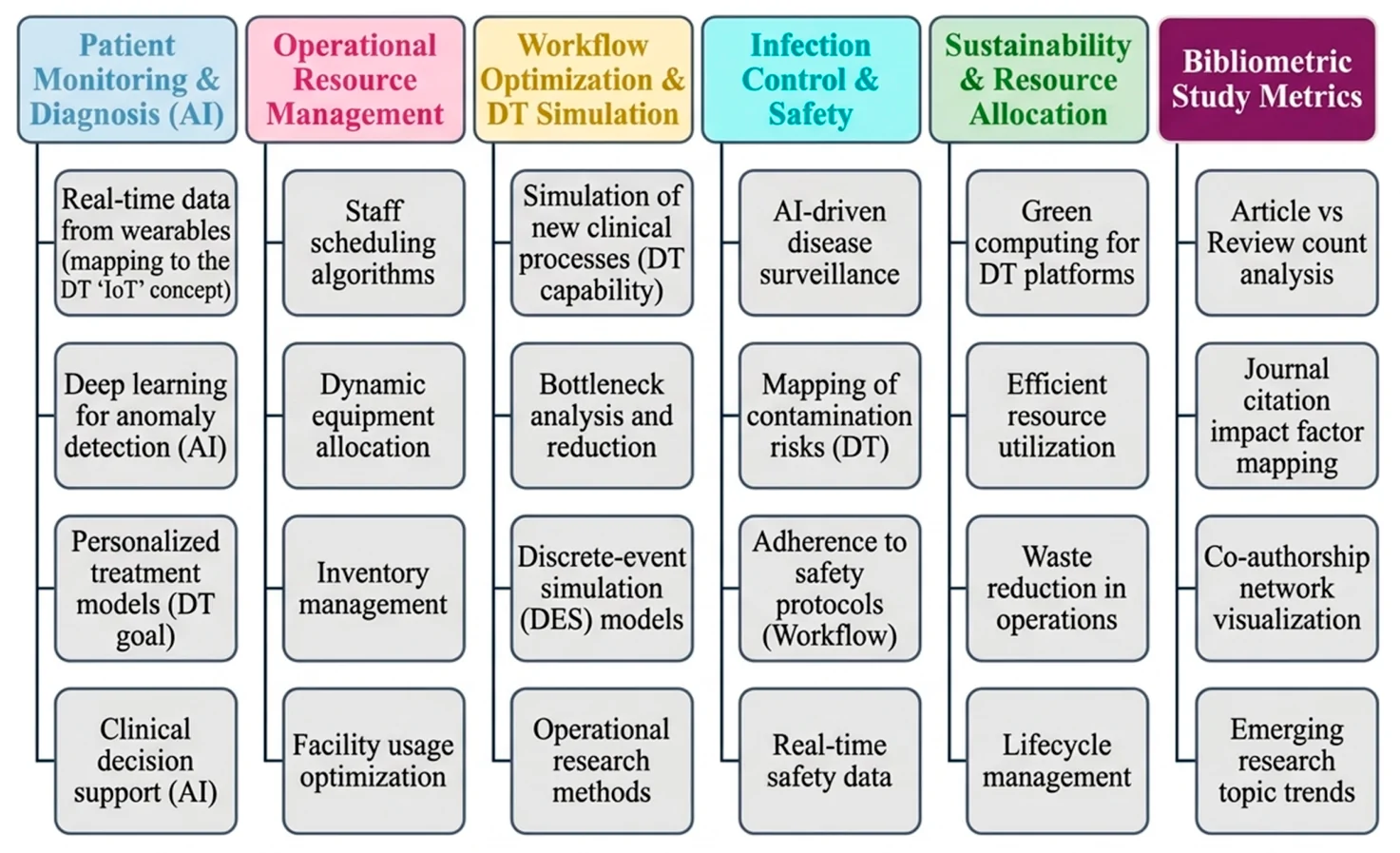
Conceptual classification of Digital Twin and AI techniques for healthcare operations.

The area of DT studies in connection with the health care sector has seen several developments since it has evolved from a technology originally based on the industrial sector, where it has been transformed into an advanced technique that facilitates hospital operation management using AI and IoT technology. Among the latest developments in the research body, it has become clear that the number of publications has greatly increased after 2021, focusing primarily on the concept of “intelligent” integration of physical and digital hospital models. Among the key drivers of green innovation are AI and IoT, which offer powerful tools for optimizing resource use, reducing waste, and promoting sustainable development. To gain a comprehensive understanding of the current state-of-the-art green technology research, this study employs a bibliometric analysis to explore scientific literature. By examining publication trends, identifying key research themes, and mapping collaborative networks, we aim to shed light on the emerging frontiers of green technology and its potential to revolutionize various sectors, as shown in Figure 6.

This systematic review was conducted and reported in accordance with the Preferred Reporting Items for Systematic Reviews and Meta-Analyses (PRISMA) 2020 guidelines. The study-selection process, including identification, screening, eligibility assessment, and final inclusion, is documented using the PRISMA 2020 flow diagram. The completed PRISMA 2020 checklist is provided as Supplementary Material. This review was not prospectively registered in a public systematic-review registry. No separate review protocol was prepared or published.

The literature search, title and abstract screening, full-text eligibility assessment, and final study selection were conducted by a single reviewer using predefined inclusion and exclusion criteria, while the final set of eligible studies and ambiguous cases were reviewed and verified by the research team. The primary objective of this work was to combine a systematic literature review with bibliometric analysis to characterize research trends and emerging developments in Digital Twins for Hospital and Healthcare Operations. Further, this review followed PRISMA 2020 guidelines. A completed PRISMA checklist will be provided as Supplementary Material.

**Figure 7:**
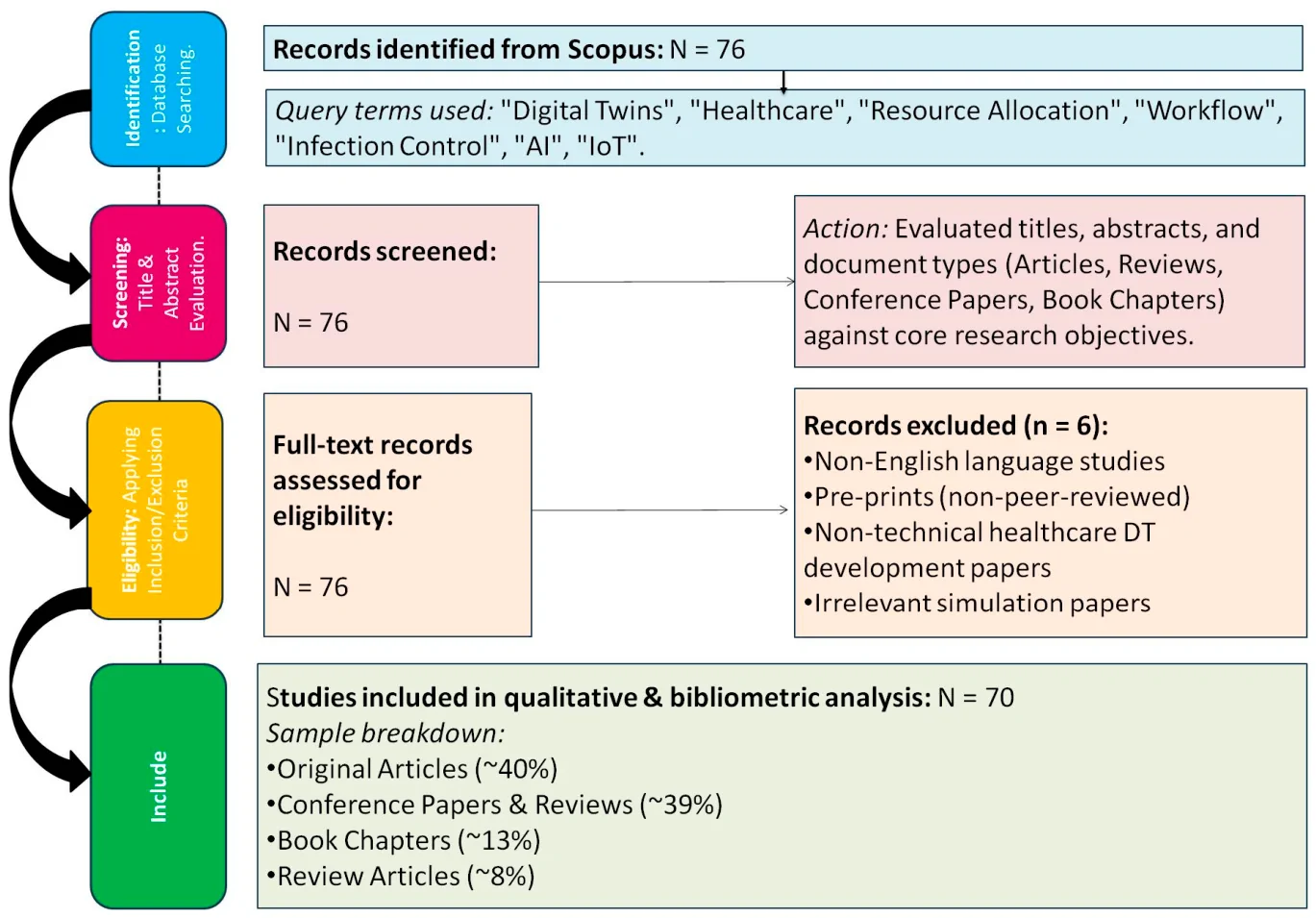
PRISMA 2020 flow diagram of the identification, screening, eligibility assessment, and inclusion of studies in the systematic review.

**Figure 8:**
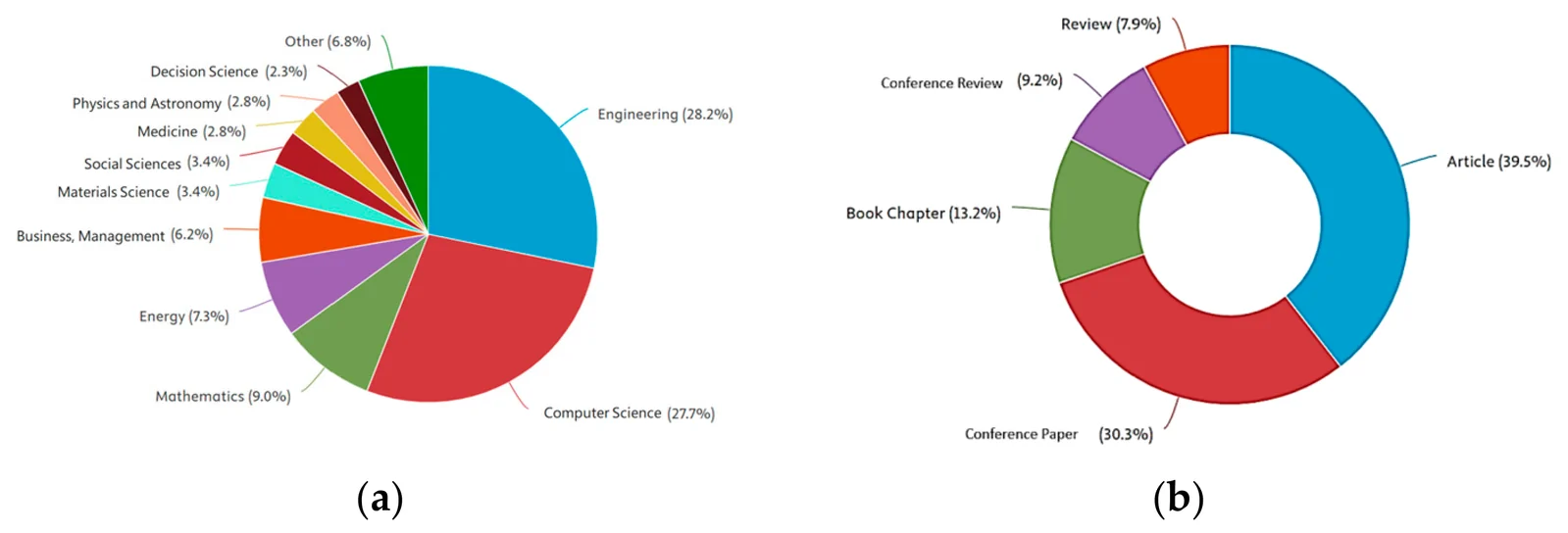
DTs for Healthcare using AI and IoT publications classification by subject area and type in (**a**) and (**b**), respectively.

**Figure 9:**
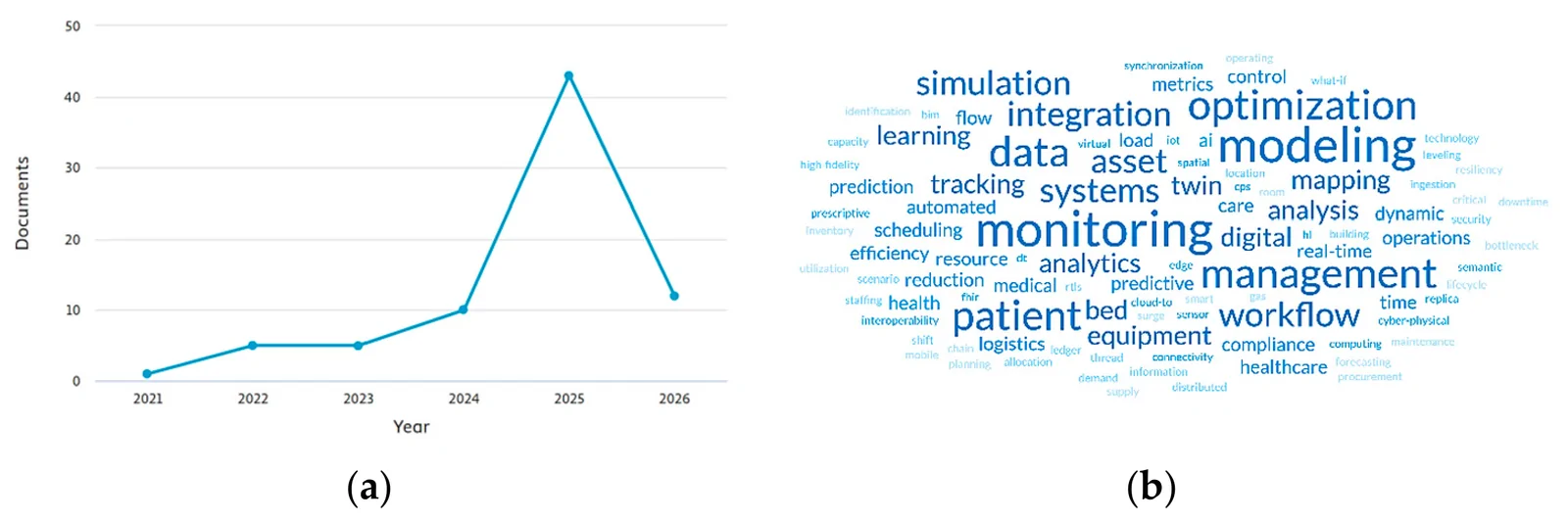
DTs for Healthcare using AI and IoT publications Scopus publications over the years (2021–2026) in (**a**) vs. the word cloud chart of important keywords used in these previous studies in (**b**).

The selection of studies followed the general principles of the PRISMA framework in Figure 7, beginning with the identification of all potentially relevant records in the database. The study was performed as follows:

(a) Determining the keyword search to use for the initial search.
(b) Streaming as seen in the PRISMA Flowchart in Figure 7.
(c) Utilization of the bibliometric analysis on the screened sample, which includes Total annual scientific contribution to this research interest in the field (Figure 8); subject-area-classified publications (Figure 8a); analysis of the type (Figure 8b).
(d) Utilization of a subsequent literature search for each key research field was discovered using the unique search phrase created for each industry research field.
(e) Review the research from Step B to ensure its applicability to each sector and to improve its validity.
(f) A co-occurrence network is used in the bibliometric analysis of each significant field to find clusters of research areas and widely used technologies. Figure 9 summarizes the key terms of Scopus publications on sustainable AI and IoT.
(g) A content analysis of the text of the selected key publications will summarize the research goals, solutions, AI/IoT components used, and whether and how the suggested solution was applied to address real-world issues.

### 3.1. Identification

A comprehensive literature search was conducted on 15 May 2026, using the Scopus database. To ensure full reproducibility, the exact executable search string applied across Article Titles, Abstracts, and Keywords (TITLE-ABS-KEY) was:

TITLE-ABS-KEY (“Digital Twin” OR “Digital Twins”) AND (“Healthcare” OR “Hospital” OR “Hospital Operations”) AND (“Resource Allocation” OR “Workflow Optimization” OR “Infection Control”) AND (“AI” OR “Artificial Intelligence” OR “IoT” OR “Internet of Things”). The initial search returned 76 documents published between 2021 and 2026. Table 1 summarizes the search strategy of this study.

### 3.2. Screening

In the screening phase, the initially identified articles were refined using subject, title, and abstract filters to ensure the relevance of the selected studies to the core research objectives.

- Subject Filter: Articles were first filtered based on their disciplinary context to retain only those aligned with the primary focus of the study. Specifically, subject areas such as Energy, Environmental Science, Engineering, Computer Science, Multidisciplinary, Arts and Humanities, and Social Sciences were selected within the Scopus database. Also, the titles and abstracts were screened for relevance. This filtering step reduced the total number of articles to 70, which were exported in BibTex and RIS format.
- Duplicates: Subsequently, the BibTeX file containing the filtered records was imported into Rstudio (2023.12.1 for Windows PC) to identify and remove duplicate entries. All duplicates were identified and removed from the database.

### 3.3. Eligibility

Review articles (7.8%) comprise the largest share, suggesting a strong emphasis on synthesizing and analyzing existing research. Articles (39.5%) represent original research papers. Conference Papers (30.3%) and Book Chapters (13.2%) indicate active participation in conferences and book publications. Conference Reviews (9.2%) represent a smaller but diverse range of document types. We have excluded non-technical research publications or non-English studies, as presented in Table 2.

### 3.4. Inclusion and Quality Assessment Criteria

Inclusion and quality assessment criteria were applied during the screening and eligibility stages of the PRISMA process. According to a Scopus analysis of publications related to Sustainable AI and IoT from 2021 to the present, the field has experienced growing research activity, while further advances are still needed to address emerging challenges and application gaps. Figure 8 and Figure 9 present the corresponding Scopus publication statistics. Studies were included if they explicitly addressed Digital Twin applications in hospitals or healthcare operations, including the integration of AI, machine learning, IoT, real-time monitoring, or predictive modeling. Eligible studies were qualitatively assessed based on the clarity of research objectives, methodological transparency, relevance to healthcare operations, and adequacy of the reported validation approach. Studies with only tangential relevance, insufficient methodological detail, or an unclear methodological focus were excluded.

Methodological Quality and Risk-of-Bias Assessment.

Because the included literature comprised heterogeneous study designs, including simulation-based studies, engineering implementations, observational investigations, and review articles, a single conventional clinical risk-of-bias instrument was not applicable across the entire corpus. Therefore, methodological quality was assessed using a structured appraisal framework informed by the principles of the Joanna Briggs Institute (JBI) critical appraisal tools and adapted to the characteristics of healthcare Digital Twin research. The appraisal considered five domains: (i) clarity of the research objectives, (ii) transparency and adequacy of the methodological description, (iii) explicit description of the Digital Twin architecture and physical–virtual synchronization mechanism, (iv) appropriateness of the validation or evaluation procedure, and (v) consistency between the reported results and study conclusions.

The initial appraisal was performed by one reviewer using the predefined criteria. The quality classifications, together with studies presenting ambiguous methodological information, were subsequently reviewed and verified by the research team. Any disagreement was resolved through discussion and consensus. No automated tools were used for methodological quality assessment.

Figure 6 shows the distribution of DTs using AI and IoT publications by subject area and document type. Engineering Field indicates a strong focus on practical applications, while Computer Science is the second-largest contributor, highlighting the fundamental role of AI and IoT technologies. Further, Energy and Business Management research indicates the relevance of these technologies in addressing energy challenges and business strategies. The remaining subject areas, including Decision Science, Mathematics, Biological Sciences, and Other, contribute to a more diverse research landscape.

Figure 9 provides insights into the evolution of Sustainable AI and IoT research over time and the key keywords associated with this field. The number of Scopus publications in this field has seen a significant increase from 2021 to 2024, followed by a great increase in 2025 and a resurgence in 2026. This trend suggests a growing interest in and investment in this research area.

**Figure 10:**
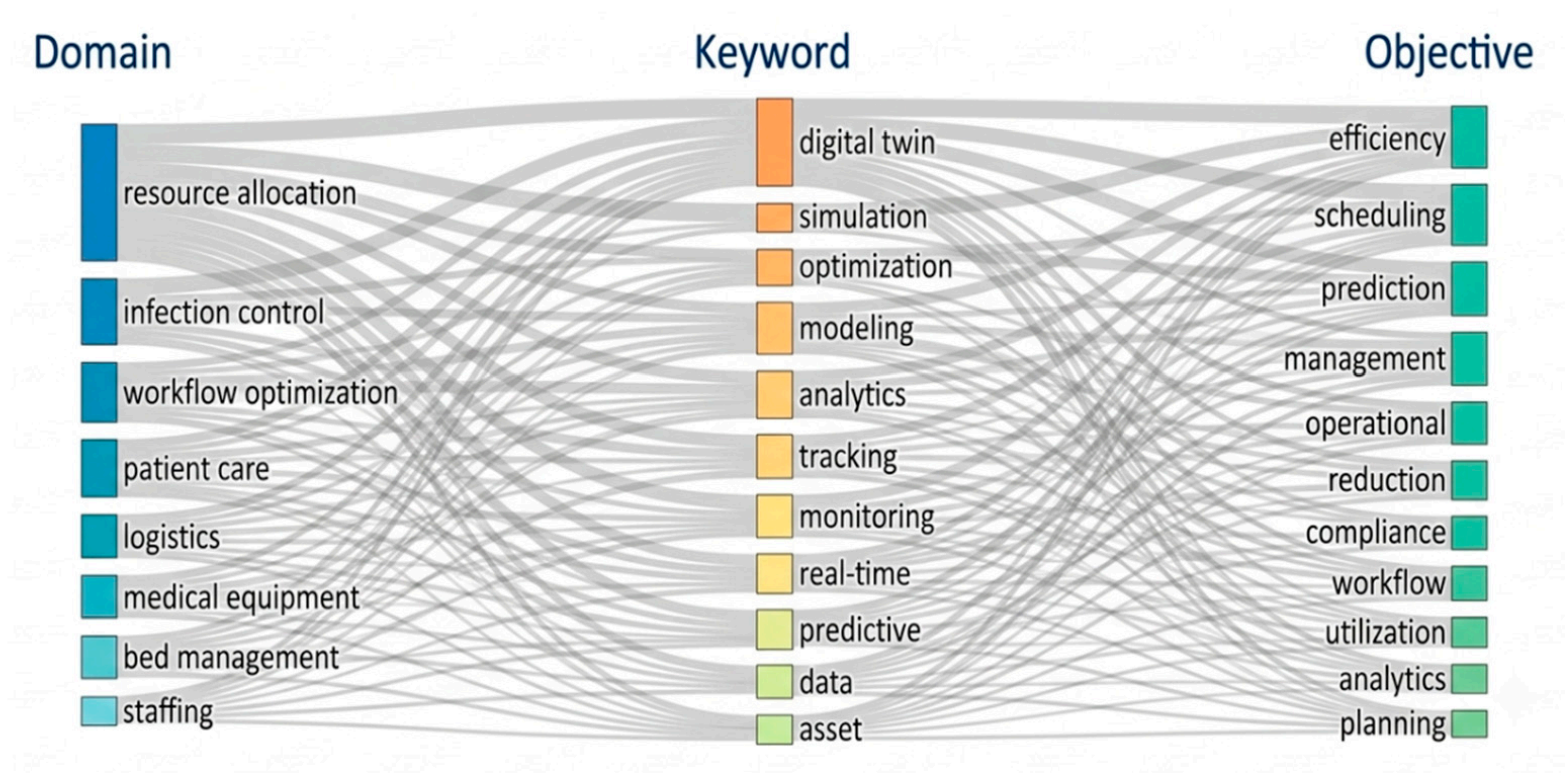
The linkage of healthcare domains, keywords, and operational objectives.

**Figure 11:**
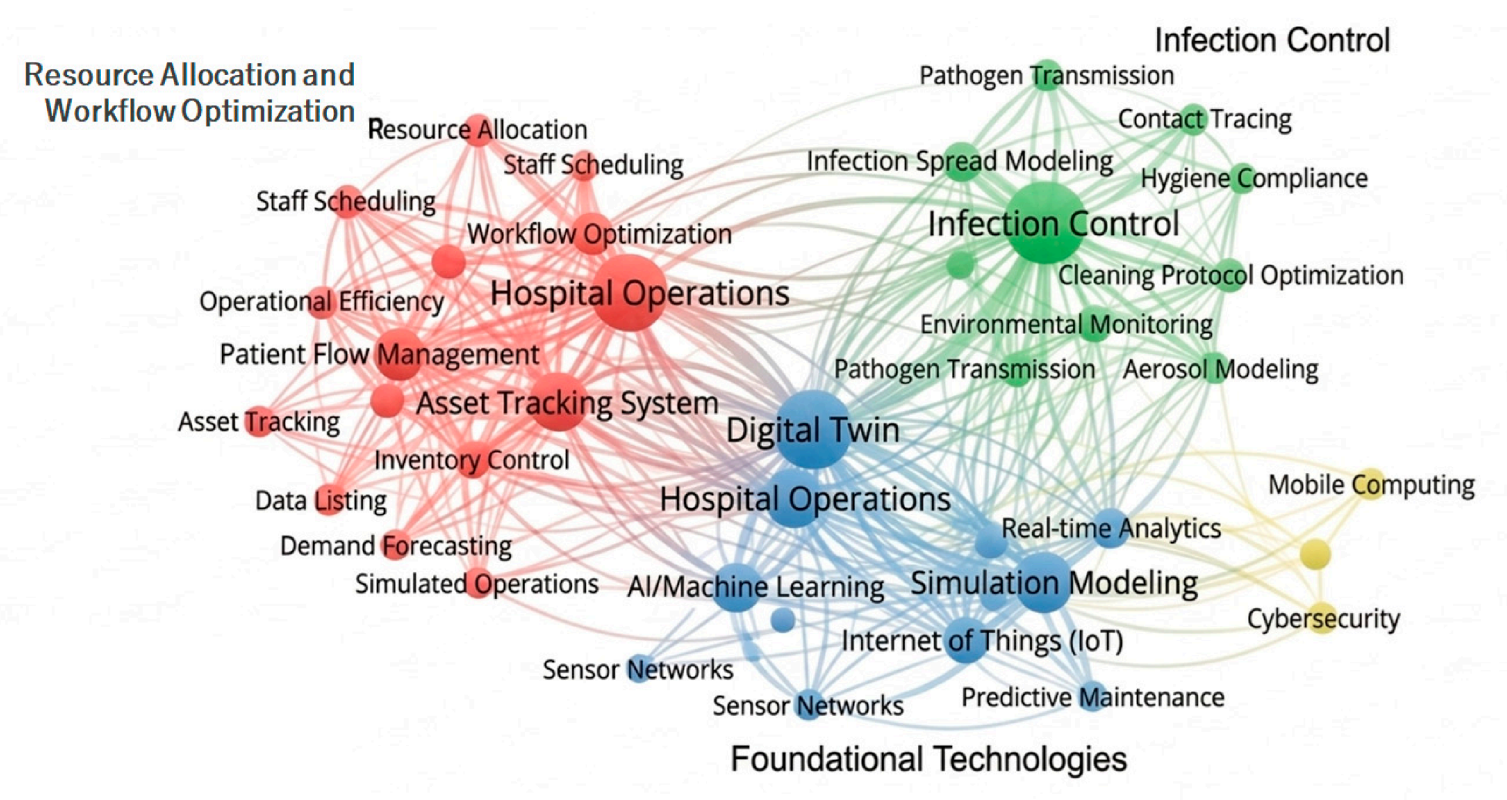
Keyword occurrence in DTs for hospital operations research.

Additionally, the word cloud analysis is limited to the most frequent keywords, potentially overlooking less common but significant terms. Prominent keywords in Figure 10 and Figure 11 include “digital twin,” “artificial intelligence,” “internet of things,” “machine learning,” “big data,” “deep learning,” “workflow,” and “medical monitoring.” These keywords collectively reflect the core themes and research priorities within the field of DTs using AI and IoT.

Each record was assessed against predefined Digital Twin criteria. Eligible studies were required to demonstrate dynamic, real-time, or continuous data synchronization between physical healthcare entities and their virtual representations. Studies limited to generic AI/IoT applications, static simulations, or non-healthcare domains were excluded. Methodological quality was evaluated using a structured appraisal framework informed by JBI principles, considering research objectives, system architecture, synchronization mechanisms, and validation approach. The initial assessment was conducted using these predefined criteria, while the final eligible studies and ambiguous cases were reviewed and verified by the research team. Of the 76 full-text reports assessed for eligibility, six were excluded because they did not satisfy the predefined Digital Twin or healthcare-operational eligibility criteria. Consequently, 70 studies were included in the final systematic review and bibliometric analysis.

It reveals a growing body of research focused on leveraging AI and IoT technologies to address sustainability challenges. The interdisciplinary nature of this field is evident from the diverse range of subject areas and document types. The increasing number of publications and the emphasis on keywords like “digital twins” and “healthcare” highlight the societal importance of this research.

### 3.5. Data Extraction and Collection Process

Data were extracted from the 70 eligible studies using a predefined structured data-extraction form developed according to the objectives of this review. For each study, the extracted information included publication year, study type, healthcare application domain, Digital Twin type, AI/ML and IoT technologies employed, data sources, synchronization mechanism between the physical and virtual entities, system architecture, validation approach, performance indicators were reported, and the principal findings and contributions.

The initial data extraction was performed by one reviewer. The extracted information, study classification, and any ambiguous or incomplete entries were subsequently reviewed and verified by the research team. Disagreements or uncertainties regarding study classification or extracted information were resolved through discussion and consensus among the authors. No automated data-extraction tools were used. Where information was not explicitly reported in the original article, it was recorded as “not reported” rather than inferred.

Assessment of Reporting Bias.

Formal statistical assessment of publication or reporting bias, such as funnel-plot asymmetry or Egger’s regression test, was not performed because the included studies were heterogeneous in terms of study design, Digital Twin architecture, healthcare application, validation strategy, and reported outcomes, and no quantitative meta-analysis was undertaken. Nevertheless, the potential for reporting and publication bias was considered qualitatively when interpreting the evidence, particularly because the review was restricted to peer-reviewed English-language publications indexed in Scopus.

## 4. Comparative Analysis with Existing Review Papers

Digital Twin technology in hospitals describes the utilization of data science and IoT to develop efficient, eco-friendly, and patient-centric services. It enhances clinical outcomes while minimizing waste and operational costs.

While recent meta-reviews have broadly discussed healthcare DTs, implementation barriers, and ethical implications, existing literature remains predominantly fragmented, either focusing on individual patient-level twins or isolated clinical departments. The primary novelty of this systematic review lies in providing a unified, hospital-level operational framework that concurrently synthesizes three interdependent pillars of hospital management: (1) real-time resource allocation, (2) dynamic infection control, and (3) workflow optimization. By evaluating how AI-and IoMT-driven DTs integrate data streams across these three dimensions, this review bridges technical architecture with operational viability and sociotechnical sustainability, offering actionable insights for scalable smart hospital deployments.

The integration of DTs into hospital and healthcare operations has garnered significant attention, emphasizing the need for key criteria to ensure effective implementation. According to Armeni et al. [16], current applications of DTs span multiple healthcare domains, including hospital operations, which highlights their potential to facilitate evidence-based decision-making and optimize workflows. The authors underscore that for DTs to realize their full potential, they must address barriers such as data interoperability, security, and integration with existing hospital systems, aligning with the broader challenges identified in digital transformation efforts.

In the context of hospital performance management, Tyagi et al. [17] advocate for a multi-criteria decision-making approach that can be adapted to evaluate and enhance hospital operations through digital twin technology. This approach emphasizes the importance of incorporating diverse performance metrics, such as efficiency, safety, and patient outcomes, which are critical criteria for the successful deployment of DTs in healthcare settings.

Augmenting this perspective, Hundal et al. [18] explore the broader landscape of digital transformation in healthcare enterprises, identifying enablers and barriers that influence the adoption of digital technologies, including DTs. They highlight that overcoming challenges such as workforce resistance, regulatory complexities, and data interoperability is essential for establishing reliable and scalable digital twin systems within hospitals.

Specific technological criteria are also emphasized in recent studies. Jiang et al. [19] demonstrate that integrating Building Information Modeling (BIM) with DTs can enhance hospital infrastructure management, particularly in real-time operational adjustments like HVAC system design. Their work underscores the importance of advanced algorithms, such as deep learning and support vector machines, for real-time data processing and predictive analytics, which are crucial for dynamic hospital operations.

Security and trust are paramount for DT deployment in healthcare. Alalisalem et al. [20] reviewed how blockchain technology can secure healthcare DTs, addressing threats related to data privacy and cybersecurity. They identify that robust security architecture, including distributed ledger technologies, is a key criterion to safeguard sensitive health data and ensure the trustworthiness of digital twin systems.

Furthermore, the applicability of DTs in managing specific health conditions, such as cardiovascular diseases, is explored by Zou et al. [21], emphasizing the need for high-fidelity modeling and real-time data integration as critical criteria for effective disease management and personalized treatment planning.

Overall, the literature converges on several key criteria for DTs in hospital and healthcare operations: interoperability of data systems, security and privacy safeguards, real-time data processing capabilities, integration with existing infrastructure, and the ability to support predictive analytics and decision-making. Addressing these criteria is essential for leveraging DTs to improve hospital efficiency, safety, and patient outcomes, ultimately facilitating a transition toward smarter, more responsive healthcare environments [18,19,20,21].

**Figure 12:**
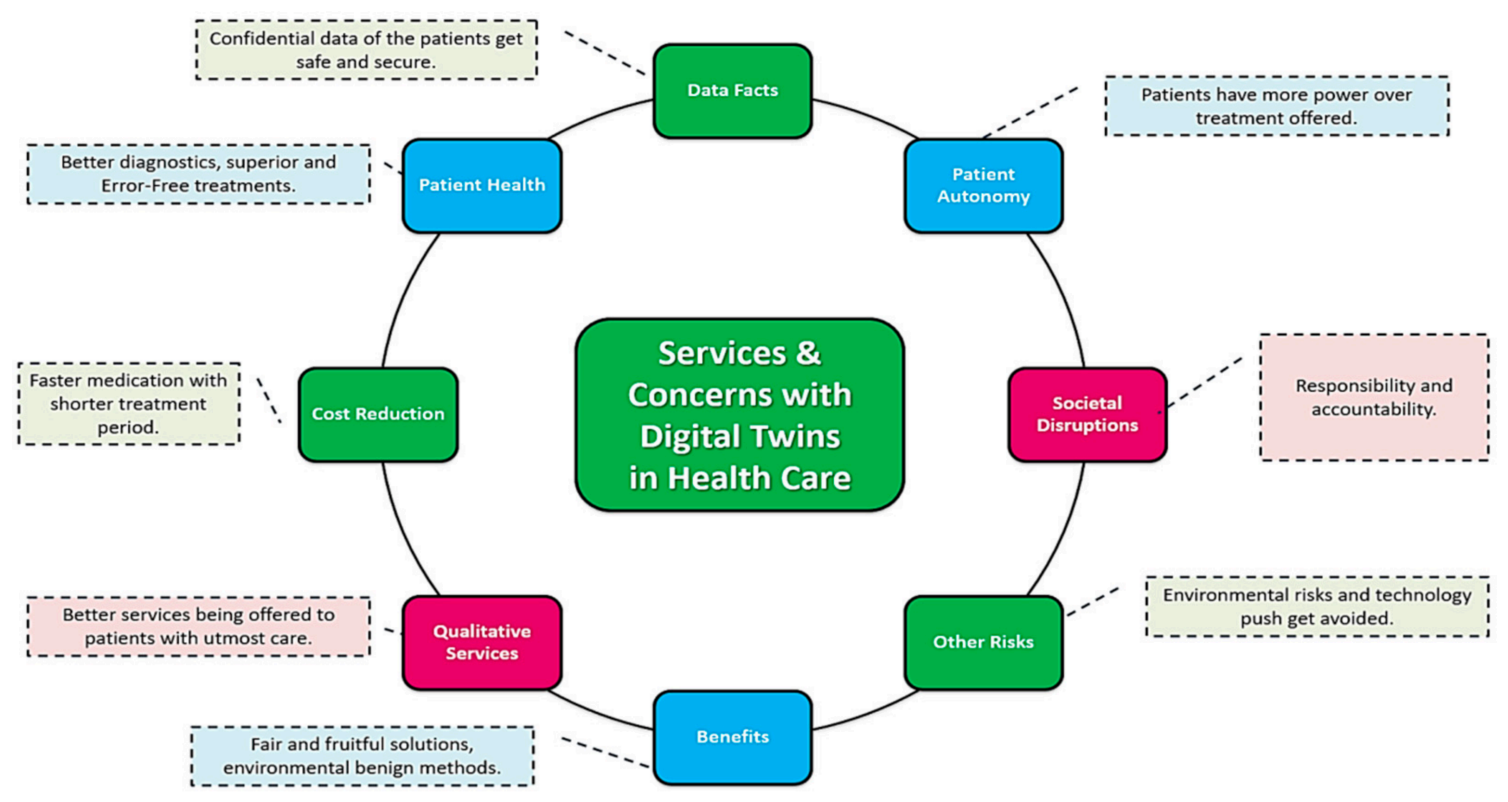
The comprehensive conceptual framework of the Services and Concerns with Digital Twins in Health Care.

The innovation of this research is demonstrated through Figure 12. This study moves away from a strict technical review towards a multi-faceted, ethical, and operational analysis, which makes it unique compared to previous research. While traditional reviews tend to emphasize solely the efficiency of algorithms used by AI and IoT, in this research, we propose a system analysis where the efficient Services, such as reduced costs, accurate treatments, and quality care, are complemented with the consideration of the important Concerns related to humans. By covering new aspects like patient choice, societal changes, and the accountability of self-regulating systems, this study addresses an obvious knowledge gap in current research. By providing a prospective vision for evaluating the sustainability of Digital Twins, not only in terms of performance but also their ability to offer fruitful solutions, this research proposes an innovative perspective for advancing smart operations of healthcare facilities.

The unique contribution of this research is that it moves away from the algorithmic approach used in literature. Unlike current literature that mainly concentrates on technical efficiency and infrastructure of DTs, this review takes an alternative approach by introducing a multi-layered framework (as shown in Figure 10). In our review, there are three unique contributions:

- Bridging Sociotechnical Divide: We combine technical efficiencies with human elements like patient rights, social considerations, and accountability of self-regulating systems.
- Ethics Considerations: The identification of the ethical issues in AI-based healthcare system helps us to make a holistic analysis which makes sure that DT system will be not only technically efficient, but also responsible and sustainable.
- Prospective Framework for Sustainability: Our framework provides a future outlook for stakeholders to determine the sustainability of DTs through balancing service delivery and human aspects—something that is missing from the current literature.

## 5. Applications of DT in Hospital and Healthcare Operations

The integration of AI and IoT allows for the creation of “living” models of healthcare facilities [22,23,24,25,26]. Table 3 summarizes recent research contributions toward optimizing hospital global risks through these technologies.

### 5.1. Infection Control and Safety

In addition, computer vision algorithms have the potential to recognize violations of sterile procedures or areas with high traffic, necessitating timely disinfection. Through airflow simulations and person-to-person contacts, DTs make it possible to “see” how infections propagate in hospitals even before an epidemic becomes apparent.

The integration of digital twin technologies into hospitals opens promising opportunities related to improving infection control in hospitals. In particular, the use of DTs provides an opportunity to replicate the status of the hospital environment, patients, and medical devices in real-time, allowing healthcare providers to monitor and optimize infection prevention processes [27]. As stated above, the relevance of the suggested methodological approaches becomes obvious when we discuss infection control and prevention in hospitals.

The existing studies indicate several benefits associated with using digital technology for infection control. For example, El-Feky et al. [28] showed the effectiveness of using PDAs in HAI detection in a NICU setting. Furthermore, various computational models could be used for analyzing the dynamics of infections, including fuzzy systems and socio-technical system analysis [29,30]. Such models allow for estimating environmental safety and predicting infection outcomes, which is beneficial for infection prevention efforts.

Cybersecurity issues related to DTs have been investigated in contemporary studies as well. For instance, Alalisalem et al. [20] provided an overview of how blockchain technology and distributed ledger technology can guarantee the security of digital twin frameworks by preserving data integrity and trustfulness in the context of real-time hospital activities. These cybersecurity solutions play a key role in protecting infection-related data flows and ensuring their confidentiality and precision, which positively impacts infection control procedures.

In addition, room-patient assignment optimization is another application area for DTs. Specifically, Danach et al. [31] suggested an epidemiology-oriented hyper-heuristic algorithm designed to optimize room assignment policies and reduce infection risks, showcasing the practicality of utilizing infection control DTs for guiding operational decision-making.

Finally, quality accreditation and management systems can benefit from digital twin capabilities. In particular, Alturbag et al. [32] demonstrated how the process of accreditation has been proven to affect infection control efficiency and patient safety positively, indicating the ability of DTs to function as monitoring and performance evaluation tools within accreditation frameworks. Moreover, the use of DTs in facilitating lean hospital practices and management control systems is discussed in contemporary literature [33].

In summary, the current literature indicates that DTs hold significant potential for advancing infection control and safety in hospitals, as seen in Table 4. They enable real-time monitoring, predictive modeling, and secure data management, all of which are essential for proactive infection prevention and operational resilience. As the field progresses, integrating these technologies with existing healthcare frameworks and ensuring cybersecurity will be critical for realizing their full benefits in safeguarding patient and staff health [27,31].

### 5.2. Workflow Optimization

The utilization of DTs in hospitals and the healthcare sector represents a novel area of research that holds great promise for improving workflow management, allocation of resources, and operational resilience. Existing literature focuses on the wide scope of applications of DTs in the field of healthcare, stressing the ability of DTs to simulate, analyze, and optimize hospital workflows.

Accordingly, Armeni et al. [34] provided an extensive literature review of DT applications in healthcare, specifically focusing on their contribution to the fields of hospital operations, precision medicine, and clinical trial designs. The authors discussed the possibilities offered by DTs to improve connected care and manage lifestyle, health, and chronic diseases, with the caveat of removing barriers to adoption of DTs. In a similar vein, Calcaterra et al. [35] analyzed existing applications of DTs in healthcare and discussed issues related to data protection and security.

As part of hospital infrastructure, Jiang et al. [19,36] proposed a method that uses BIM and DTs to innovate heating, ventilation, and air-conditioning design in hospital construction. Specifically, the authors use machine learning algorithms for the prediction of pedestrian flows and discuss the possibilities offered by DTs in management and operational efficiency.

Optimization of operational workflows in hospitals can be achieved by Silva-Aravena et al. [37], who applied digital twin technology with reinforcement learning algorithms for optimizing scheduling of MRIs in complex hospitals. The approach is designed to improve satisfaction of patients with hospital services and increase effectiveness of care, through increasing resource utilization and minimizing waiting times. These studies highlight the capabilities of DTs for improving the efficiency of hospital operations.

The problem of resilience of health institutions during crises is discussed by Lu et al. [38]. This study examines a possible application of blockchain-empowered dynamic DTs for building an interconnected network of hospitals that can operate effectively during pandemics by integrating information and allocating resources according to the severity of the situation within particular regions.

Despite many applications being concerned with infrastructural issues, there are other factors that need to be considered for effective development of digital twin solutions. Cakir et al. [39] discussed problems associated with DTs’ synchronization and interoperability across networks. The authors provide metrics for measuring synchronization performance and the effectiveness of interoperability.

In conclusion, the current body of scientific literature indicates that Digital Twins (DTs) have substantial potential to transform hospital operations and the broader healthcare sector through real-time monitoring, predictive modeling, and adaptive decision-making processes [36,37]. Table 5 summarizes DTs in Healthcare systems workflow.

Overall, the literature suggests that the effective integration of Digital Twins into healthcare workflows depends on reliable network synchronization and accurate alignment between virtual models and real-world measurements. Moving beyond static queueing models toward dynamic, AI-enabled digital replicas can support adaptive scheduling, reduce operational bottlenecks, and enhance continuity of care during unexpected increases in patient demand [36,37,38].

### 5.3. Resource Allocation

With the use of deep learning models, the ability to forecast the “traffic flow” in emergency departments can help to allocate nurses and doctors to more crowded spots where the resources could be used most effectively to reduce both waiting time for patients and the level of exhaustion of healthcare workers.

The implementation of DTs in resource allocation practices within hospitals is an important yet relatively new concept that holds a lot of promise when it comes to improving hospital efficiency and enhancing patient experience. Ghatti et al. [40] discussed the state-of-the-art digital twin solutions in healthcare by stressing the importance of solving associated security and ethical questions, namely privacy and data protection, to create a sustainable resource allocation system.

Further, the idea of using blockchain technology and integrating it into DTs to improve healthcare management and resource allocation at the municipal and even city-wide level is presented in the work by Lu et al. [41]. The authors present the theoretical basis for predicting medical resource needs with the help of analyzing patient trajectories and combining information from various HISs.

The context of DTs within the field of healthcare is also provided by Ghatti et al. [40], who stress the significance of addressing the issues of security, privacy, and ethics while developing resource allocation frameworks. The role of applying AI to supply chain management and, specifically, resource allocation, in this case, has been analyzed by Adedunjoye et al. [42].

Mutashar et al. [43] suggested that a cloud-based digital twin, combined with IoT technology, can assist in managing resources efficiently and improving workflow in emergency departments in hospitals. As shown in their case study, DTs can prove useful to hospitals in the allocation of resources, particularly in their essential functioning parts.

In a similar manner, Silva-Aravena et al. [37,44] examined the application of DTs together with reinforcement learning to enhance MRI scheduling in hospitals. The proposed approach highlights the potential of DTs to contribute to the process of optimization by facilitating adaptive allocation of resources to boost customer satisfaction and improve results. These examples reveal that AI methods may be applied effectively when developing digital twin models.

Further, Gerstmeier et al. [45] examined the issues related to resource and staffing benchmarking in a tertiary hospital to set up the benchmarks in terms of resource allocation in real-life situations. Therefore, the need to establish benchmarks based on data obtained during the operational processes of a hospital becomes evident.

Regarding the problems related to security and scalability, the paper by Zhao et al. [46] introduced a blockchain-based sharding framework for medical DTs, which allows optimizing resource allocation and cluster head selection while performing spectrum management tasks to achieve maximum transaction throughput and ensure data security. It is worth mentioning that the proposed adaptive resource allocation framework shows that blockchain technology can be used for secure resource management within the networks of healthcare DTs.

In summary, the literature collectively indicates that DTs, especially when integrated with AI and blockchain technologies, hold significant promises for optimizing resource allocation in hospital and healthcare operations. These systems enable real-time monitoring, predictive demand forecasting, and secure data sharing, which are essential for efficient resource management in complex healthcare environments; see Table 6. However, addressing security, privacy, and interoperability remains a critical ongoing challenge to fully realize their potential [41,42,43,44,45,46].

## 6. Performance Evaluation, Metrics and Benchmarking

The evaluation of DTs in healthcare operations requires a multi-layered approach that bridges technical AI performance with tangible hospital outcomes. As of 2026, the literature identifies five critical dimensions for assessing the effectiveness of these systems in resource allocation, infection control, and workflow optimization.

### 6.1. Operational Performance Metrics

Operational metrics primarily reflect the macro-level efficiency of the hospital ecosystem. Digital Twins have shown considerable potential to improve hospital throughput and patient flow [47,48,49,50]. (i) Delay Time Reduction: DT implementation has been associated with reductions of 20–40% in emergency department (ED) waiting times [43]. (ii) Patient Throughput: By enabling optimization of block scheduling and workforce allocation, virtual replicas can contribute to a 10–20% improvement in overall patient throughput [37,47]. (iii) Predictive Accuracy: Surge-capacity planning evaluates the ability of the Digital Twin to anticipate fluctuations in demand; for instance, some models have successfully predicted winter surges approximately one week before their actual occurrence [46,51].

### 6.2. AI Evaluation Metrics

The “intelligence” of the Digital Twin is evaluated through metrics that assess the underlying Machine Learning and Deep Learning models [51,52,53]. (i) Predictive Precision: Evaluated by minimizing the loss function between predicted future states and actual observed states. (ii) Convergence and Latency: For real-time applications, metrics include convergence speed of reinforcement learning agents (e.g., MAPPO) and network latency, which has seen reductions of 20–33.4% in optimized MEC-DT frameworks. (iii) Energy Efficiency: A critical metric for green healthcare, where DT-driven offloading strategies aim for a 10–53.8% improvement in power usage.

### 6.3. Healthcare Efficiency Indicators

These indicators measure the direct impact on clinical resource utilization and cost-effectiveness [53,54,55,56,57,58]. (i) Bed Utilization Rate: Monitoring real-time occupancy and predicting specialty mismatches to ensure high-priority patients (e.g., from Critical Care) are placed correctly. (ii) Length of Stay (LOS): Evaluating the twin’s ability to identify bottlenecks that prolong patient stays, thereby improving bed turnover. (iii) Infection Control Compliance: Metrics include air quality improvements (up to 20%) and tracking pathogen diffusion simulations in high-risk zones like operating theaters.

### 6.4. Benchmark Datasets and Simulation Environments

Standardization remains a challenge, but several foundational environments have emerged for benchmarking DT performance. (i) Simulation Platforms: Any Logic [59] is widely utilized for its multi-method modeling capabilities (agent-based and discrete-event), allowing for hospital-level “stress tests”. (ii) Large-Scale Simulators: Frameworks like ALMA [60] (Aotearoa’s Large-Scale Multi-Agent simulator) provide benchmarks using synthetic populations with over 80 attributes to simulate disease spread and resource demand. (iii) Integrative Data Sources: Benchmarking often relies on the fusion of Electronic Health Records (EHRs), real-time IoT sensor streams, and Building Information Modeling (BIM) data.

The key benchmark datasets/data sources employed in DT medical domains include:

EHR and Critical Care MIMIC-IV (Medical Information Mart for Intensive Care) [61]: This deidentified clinical database available through PhysioNet [62] contains detailed electronic health records, lab results, medication administration records, and ICU vital signs. It is one of the most comprehensive benchmarks used in developing holistic patient twins at the level of processes and systems.

- INSPIRE [63]: This PhysioNet [62] dataset is extensively used in the analysis of perioperative medicine and monitoring the dynamics of the workflow in real time.
- Cardiovascular Digital Twins UK Biobank Cardiac Meshes [64]: Patient-specific meshes of more than 1400 adult heart models extracted from cardiovascular MRIs. It is one of the key benchmarks for 3D electro-mechanical modeling in different demographics.
- ACDC Dataset (Automated Cardiac Diagnosis Challenge) [65]: Benchmark for deep learning-based assessment of heart function and movement representations using 4D cine cardiac MRI volumes.
- Neurological & Biosignal Analysis MIT-BIH Arrhythmia Database [66]: Key PhysioNet database serving as the gold-standard benchmark for electrocardiogram (ECG) tracking and anomaly detection algorithms.
- System and Metabolic Twins (Diabetes)UVA/Padova T1DM Simulator Dataset [67,68]: Very standardized benchmark representing glucose metabolism. It is extensively used in testing and validating artificial pancreas models and closed-loop insulin delivery algorithms.

## 7. Challenges and Future Directions

The integration of DTs into hospital and healthcare operations presents a promising avenue for enhancing patient care, operational efficiency, and resource management [69,70]. Current methods and applications of DTs in healthcare are expanding, yet significant challenges related to security and ethical considerations persist. Privacy concerns and data protection are critical issues that need to be addressed to facilitate broader adoption of digital twin technology in clinical settings.

The transformative potential of DTs in hospital practices, highlighting their role in improving workflow efficiency and patient care. Through case studies and literature review, the paper illustrates how DTs are revolutionizing traditional hospital operations, although it also implicitly points to challenges such as integration complexity and the need for robust data governance frameworks.

Also, the integration of artificial intelligence, big data, and DTs is accelerating, which underscores the importance of developing advanced simulation methods that can support dynamic and complex healthcare environments. However, this evolution also introduces challenges related to technological interoperability and the need for standardization.

Further, the broader impact of technological innovations in healthcare emphasizes the importance of addressing implementation challenges such as sustainability, inclusivity, and ethical considerations. These factors are particularly relevant for DTs, which require extensive data sharing and integration across diverse healthcare systems, raising concerns about equitable access and ethical data use.

Moreover, data-driven approaches to improve patient flow and service efficiency, highlighting the role of predictive analytics, AI, and digital dashboards. The study underscores the importance of governance, interdisciplinary collaboration, and policy support in sustaining operational improvements facilitated by digital twin technologies, pointing to the need for comprehensive frameworks to manage these complex systems effectively. Looking towards future directions, the development of digital-intelligent frameworks for precision health management, especially in chronic disease prevention and control. They identify translational and ethical challenges that must be addressed to embed DTs within population health strategies, emphasizing the importance of data integration, model accuracy, and ethical considerations.

Finally, federated learning and next-generation IoMT are enablers of decentralized digital twin applications, allowing healthcare entities to collaborate without compromising sensitive data. This approach addresses privacy concerns and opens new avenues for scalable, secure digital twin implementations, although it also introduces open issues related to technological integration and network security.

One limitation of this review is its reliance on Scopus as the primary database for corpus retrieval. Although Scopus provides broad peer-reviewed coverage across computer science, engineering, and healthcare-related disciplines, additional databases such as PubMed/MEDLINE and Web of Science may capture further clinically focused studies. Future updates of this review could therefore broaden the search strategy to include specialized clinical and multidisciplinary databases, thereby improving the comprehensiveness of the evidence base.

**Figure 13:**
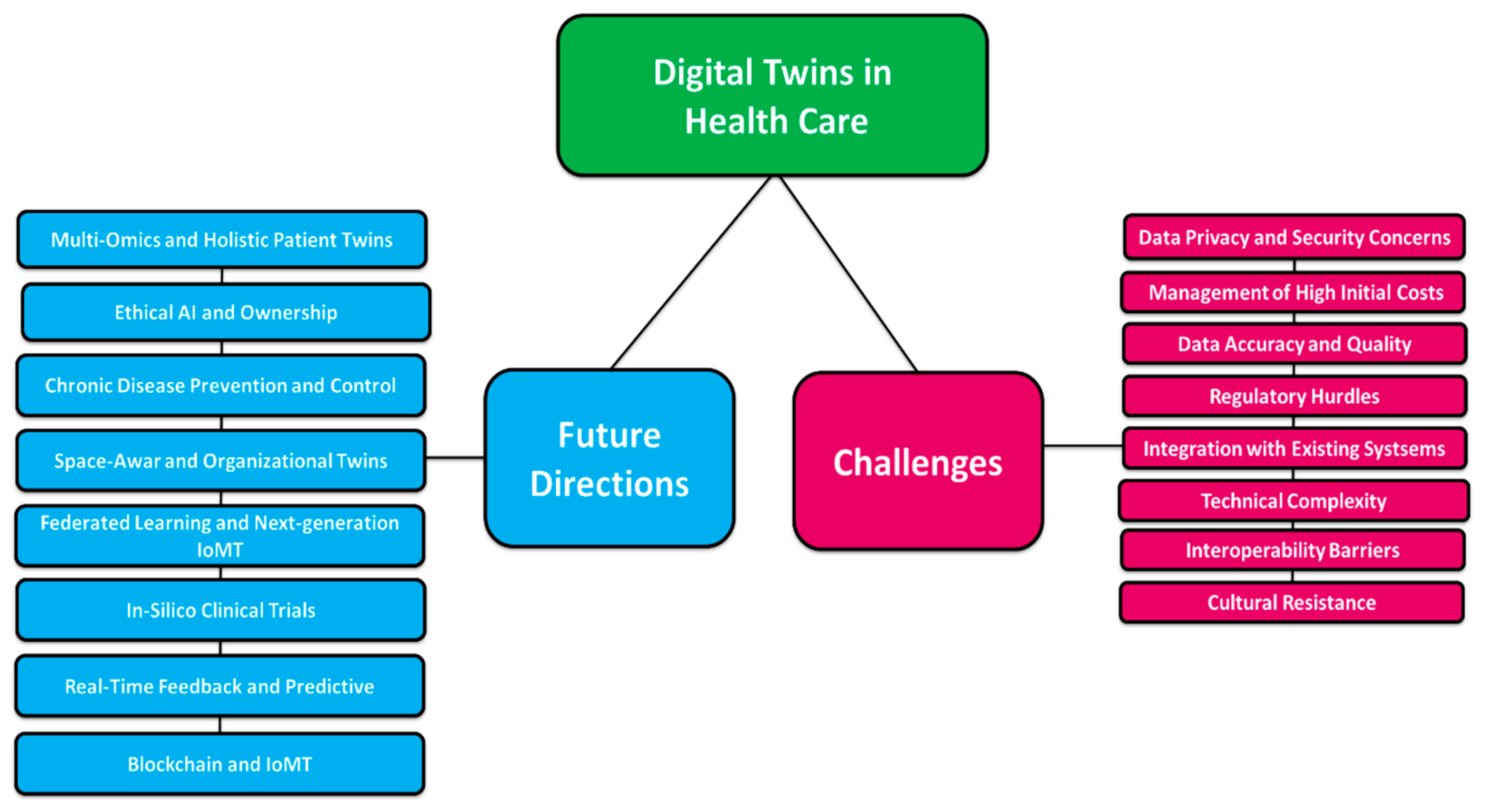
Challenges and future directions of digital twins in the healthcare sector.

In summary, while DTs hold significant promises for revolutionizing hospital and healthcare operations, challenges related to security, ethical considerations, interoperability, and governance remain prominent as presented in Figure 13. Future research is directed towards enhancing data privacy, developing standardized frameworks, and leveraging emerging technologies such as federated learning and IoMT to realize the full potential of DTs in healthcare settings. Future research should focus on Decentralized AI and Blockchain to ensure data security while maintaining the transparency required for operational twins.

## 8. Conclusions

The concept of DTs presents a fundamental transformation in the way healthcare institutions function. It enables hospitals to utilize their most important assets, which include time, equipment, and personnel, through proactive simulations rather than reactive management practices. The shift towards “Smart Hospitals,” which harness the power of artificial intelligence and the Internet of Things, is not simply an innovation in technology; it is a crucial step towards ensuring the viability of healthcare infrastructure around the world.

However, when the DTs technology is combined using the synergistic effect of AI and IoT, it leads to a breakthrough in the modern healthcare management system. This research shows that DTs have moved beyond the theory and became necessary means of moving from reactive to proactive and resilient environments for hospitals. High-fidelity DTs of patients, medical equipment, as well as an entire hospital infrastructure will help healthcare providers model possible situations and allocate resources in a safe manner. The review of the literature and the theoretical framework developed in this study revealed three main aspects of including the impact of DT on healthcare, Improved Infection Control by applying computer vision and real-time simulation technologies to predict and control the spread of pathogens before the situation becomes an epidemic, a Green Infrastructure through IoT-BIM integration; and the management of energy usage and environmental safety in the hospital structure. Yet, several obstacles will have to be overcome to bring these applications to fruition. Many issues related to data privacy, cybersecurity, and interconnectivity are still prevalent. It is crucial that future research focuses on creating security measures using blockchain technologies and performance metrics to guarantee both efficiency and security of these digital mirrors. In summary, as the technology behind DT advances, it holds immense potential to transform the field of healthcare both through improved health care delivery and better patient outcomes through precision medicine and through its role as the foundation of our hospital infrastructure.

## Figures and Tables

**Table 1:** Search Strategy for this study.

Item	Description
Database	Scopus Database
Exact Search Query	TITLE-ABS-KEY (“Digital Twin” OR “Digital Twins”) AND (“Healthcare” OR “Hospital” OR “Hospital Operations”) AND (“Resource Allocation” OR “Workflow Optimization” OR “Infection Control”) AND (“AI” OR “Artificial Intelligence” OR “IoT” OR “Internet of Things”)
Time Period	2021–2026
Document Types	Original Articles (39.5%), Conference Papers (30.3%), Book Chapters (13.2%), Conference Reviews (9.2%), Review Articles (7.8%)
Language	English
Core DT Criterion	Must exhibit continuous or bidirectional real-time data synchronization
Final Included Papers	70

**Table 2:** Inclusion and exclusion criteria for this study.

Inclusion Criteria	Exclusion Criteria
Healthcare DT papers	Non-Technical healthcare DT development studies
Peer-reviewed studies	Pre-prints
English papers	Non-English studies
AI/IoT-integrated DTs	Irrelevant simulation papers

**Table 3:** Applications for DTs and AI for Healthcare Operations.

Work	Category	Contribution
Workflow Optimization	Machine Learning	Predicting patient discharge times to improve bed turnover.
Infection Control	Computer Vision	Monitoring hand hygiene compliance and PPE usage in real time.
Resource Allocation	Deep Learning	Optimizing the supply chain for critical medications and ventilators.
Asset Tracking	IoT & Sensors	Real-time location systems (RTLS) for tracking mobile medical equipment.
Facility Management	Virtual Agents	AI-driven assistants for navigating patients through hospital corridors.

**Table 4:** Summary of DTs in Infection Control and Safety.

Author(s)	Key Technologies & Focus	Application Domain	Key Findings & Contributions
El-Feky et al. [28]	PDAs (Personal Digital Assistants)	NICU Setting	Demonstrated the effectiveness of portable digital tools in detecting Healthcare-Associated Infections (HAIs).
Fuzzy/Socio-technical Models [29,30]	Fuzzy Systems, System Analysis	Environmental Safety	Used to analyze infection dynamics and predict outcomes to enhance prevention efforts.
Alturbag et al. [32]	DT, Monitoring Tools	Quality Accreditation	Shows how DTs act as performance evaluation tools to improve patient safety and accreditation efficiency.
Alalisalem et al. [20]	Blockchain, Distributed Ledger	Data Integrity	Investigates how blockchain ensures the security and confidentiality of real-time infection-related data flows.
Lean Practice Studies [33]	DT, Management Control	Lean Hospital Practices	Explores using DTs to facilitate waste reduction and process optimization within hospital management.
Danach et al. [31]	Hyper-heuristic Algorithms	Room-Patient Assignment	Optimizes room policies to minimize infection risks through epidemiology-oriented decision-making.
Computer Vision (General)	Computer Vision, Airflow Simulation	Surveillance &Transmission	Enables “seeing” sterile procedure violations and simulating airborne/contact propagation of pathogens.

**Table 5:** Summary of DTs in Healthcare systems workflow.

Author(s)	Key Technologies & Focus	Application Domain	Key Findings & Contributions
Armeni et al. [34]	DT, Connected Care	Hospital Operations & Precision Medicine	Explores DTs in managing chronic diseases and clinical trials; identifies barriers to adoption as a primary concern.
Calcaterraet al. [35]	DT	Data Security	Analyzes existing applications with a specific focus on data protection and cybersecurity risks.
Jiang et al. [36]	BIM (Building Information Modeling), DT, ML	Hospital Construction & HVAC	Uses machine learning to predict pedestrian flows and optimize ventilation/heating for operational efficiency.
Silva-Aravena et al. [37]	DT, Reinforcement Learning	Diagnostic Scheduling (MRI)	Focuses on resource utilization and minimizing patient wait times through optimized scheduling.
Lu et al. [38]	Blockchain-empowered Dynamic DT	Pandemic Resilience	Proposes an interconnected network of hospitals to allocate resources regionally during health crises.
Cakir et al. [39]	Networked DTs	Synchronization & Interoperability	Provides metrics to measure how well DTs sync and communicate across different networks.

**Table 6:** Summary of DTs in Resource Allocation.

Author(s)	Key Technologies & Concepts	Primary Application/Focus	Key Findings & Contributions
Ghatti et al. [40]	Digital Twins (DT), AI	General Healthcare Systems	Stresses the necessity of solving security, privacy, and ethical issues to create sustainable resource allocation.
Lu et al. [41]	DT, Blockchain, Patient Trajectory Analysis	Municipal/City-wide Management	Integrating HIS data with blockchain and DTs to predict medical needs across a city-wide level.
Adedunjoye et al. [42]	AI	Supply Chain Management	Analyzes the role of AI in optimizing supply chains and specific resource allocation tasks.
Mutashar et al. [43]	Cloud-based DT, IoT	Emergency Departments (ED)	Uses IoT to manage workflow and allocate resources efficiently within essential hospital departments.
Silva-Aravena et al. [37,44]	DT, Reinforcement Learning	MRI Scheduling	Facilitates adaptive allocation of diagnostic resources to improve results and patient satisfaction.
Gerstmeier et al. [45]	Benchmarking, Operational Data	Tertiary Hospitals	Establishes the need for real-life staffing and resource benchmarks based on actual operational data.
Zhao et al. [46]	DT, Blockchain (Sharding)	Network Security & Spectrum Management	Introduced a framework to maximize transaction throughput and ensure secure resource management in DT networks.

## Data Availability

The data supporting the findings of this systematic review were derived from publicly available published studies. The search strategy, eligibility criteria, study-selection process, and characteristics of the included studies are provided within the article.

## Supplementary Materials

The following supporting information can be downloaded at: https://www.mdpi.com/article/10.3390/bioengineering13091072/s1. PRISMA 2020 Checklist [71].
